# Long-term response to mood stabilizer treatment and its clinical correlates in patients with bipolar disorders: a retrospective observational study

**DOI:** 10.1186/s40345-017-0093-5

**Published:** 2017-07-09

**Authors:** Sung Woo Ahn, Ji Hyun Baek, So-Yung Yang, Yongkang Kim, Youngah Cho, Yujin Choi, Kounseok Lee, Taesung Park, Kyung Sue Hong

**Affiliations:** 1 Department of Psychiatry, Sunkyunkwan University School of Medicine, Samsung Medical Center, Seoul, Republic of Korea; 2 Department of Statistics, Seoul National University, Seoul, Republic of Korea; 3 Department of Psychiatry, Seoul National University Bundang Hospital, Seongnam, Republic of Korea; 4 Center for Clinical Research, Samsung Biomedical Research Institute, Seoul, Republic of Korea; 5 Department of Psychiatry, St. Andrew’s Hospital, Icheon-si, Republic of Korea

**Keywords:** Bipolar disorders, Treatment response, Alda scale, Lithium, Valproate

## Abstract

**Background:**

The efficacy and utility of long-term prophylactic treatment in patients with bipolar disorders (BDs) have not been fully explored. This study aims to estimate the long-term clinical response of patients with BDs to mood stabilizer treatment and to identify the clinical factors associated with that response.

**Methods:**

The study subjects consisted of 80 patients with bipolar I or bipolar II disorder who had been receiving treatment with lithium and/or valproate for more than 2 years at a single bipolar disorder clinic. The long-term response to the best treatment option based on treatment algorithms was evaluated using the Alda scale. Clinical characteristics were evaluated on a lifetime basis. Patients were classified into two response groups based on frequentist mixture analysis using the total Alda scale score.

**Results:**

Thirty-four percent of the patients were good responders, with a total Alda score of 5 or higher. The treatment response rate did not differ between the lithium and valproate groups, but lithium and valproate combination therapy was associated with poorer response. The number of previous mixed episodes was associated with a worse response (*p* = 0.026). Of individual symptoms, delusions during manic episodes (*p* = 0.008) and increased appetite (*p* = 0.035) during depressive episodes were more common in moderate/poor responders than in good responders. Co-morbid anxiety disorders were more frequently observed in the moderate/poor response group (*p* = 0.008).

**Conclusions:**

Psychotic, mixed, and atypical features of BDs were found to be correlated with long-term treatment outcomes. Lithium and valproate showed similar efficacy but moderate/poor responders preferred to use polypharmacy.

**Electronic supplementary material:**

The online version of this article (doi:10.1186/s40345-017-0093-5) contains supplementary material, which is available to authorized users.

## Background

Bipolar disorders (BDs) are a group of chronic psychiatric illnesses with diverse clinical courses composed of combinations of (hypo)manic and depressive episodes (Sadock et al. 2014). Although a number of guidelines have been established for the pharmacotherapy of BDs (Yatham et al. 2013; Jeong et al. 2015; Goodwin 2009), long-term drug response and its clinical correlates in BD patients receiving standard clinical care have not been well explored. Considering the wide variation between individuals in the manifestation of illness, including biphasic and recurring courses, difficulties in defining outcomes should be expected. Another obstacle to the investigation of long-term response is variability in drug options and changes in the drugs that are taken during the course of treatment (Ghaemi et al. 2006; Baek et al. 2014).

The criteria for long-term response have been defined in various ways. One or a few isolated variables, such as time to recurrence, reduction of episode frequency, length, or severity, and reduction of the time spent in the hospital, have frequently been used in previous studies (Kleindienst et al. 2005). These simple variables, however, do not reflect the diversity of treatment courses observed in natural clinical settings. Global assessment of functioning (GAF) (American Psychiatric Association 2000) and the clinical global impressions scale for use in bipolar illness (CGI-BP) (Spearing et al. 1997) have also been used to evaluate long-term efficacy (Kusalic and Engelsmann 1998; Post et al. 2010). However, these scales are generally only appropriate for assessing a patient’s state at a single cross-sectional time point. A scale specifically tailored to the retrospective assessment of prophylactic lithium response in BDs was introduced by Alda and colleagues (Grof et al. 2002) (the retrospective criteria of lithium response in research subjects; the Alda scale), and has been widely used in genetic studies on lithium response (Grof et al. 2002; Passmore et al. 2003; Hou et al. 2016; Chen et al. 2014; Squassina et al. 2011). As the scale considers confounding factors, such as polypharmacy, compliance, and disease course before administration of medication, it can be adapted to various clinical cases and settings involving long-term treatment.

A number of previous studies investigated potential predictors of prophylactic lithium treatment in BDs. According to a systematic review by Kleindienst and colleagues (2005), depression-mania episode sequence, earlier onset of illness, a high number of previous hospitalizations, and continuous cycling seem to be associated with poor outcomes. In later studies, psychotic symptoms, inter-episodic residual symptoms, mixed episodes, and rapid cycling (Backlund et al. 2009; Pfennig et al. 2010; Silva et al. 2016) were identified as predictors of poor long-term response to lithium. A positive correlation of long-term response with hyperthymic temperament (Rybakowski et al. 2013) and typically episodic course of illness with earlier onset (Garnham et al. 2007) were also reported. Comorbid anxiety disorders and alcohol abuse/dependence were associated with poor prophylactic efficacy in both early and recent studies (O’Connell et al. 1991; Young et al. 1993; Kliwicki 2014). Controversial results were generated regarding bipolar I disorder (BD-I) vs. bipolar II disorder (BD-II) (Kleindienst et al. 2005; Garnham et al. 2007; Rybakowski 2014) and a family history of mood disorders (Kliwicki 2014; Mendlewicz et al. 1973; Maj et al. 1985; Misra and Burns 1977; Coryell et al. 2000).

In contrast to the lithium response, studies on long-term response to the broader category of mood stabilizers are limited. A recent study evaluating the prophylactic efficacy of lithium, valproate, and carbamazepine reported an association between the likelihood of relapse and a mixed episode and the total number of manic or depressive symptoms prior to the observational period (Peselow et al. 2015). In naturalistic observational studies of the Stanley Foundation Treatment Outcome Network that assessed the prospective outcomes of BD patients receiving various combinations of pharmacologic treatments, family history of drug abuse, history of childhood abuse, a greater number of prior episodes, and comorbid substance abuse were associated with poor outcome (Post et al. 2010; Nolen et al. 2004). To the best of our knowledge, long-term studies investigating specific predictors of treatment response to valproate are lacking (Carvalho and McIntyre 2015).

According to a recent multi-center investigation of prescription patterns in Korea, valproate is more commonly used than lithium, and polypharmacy is used in 80.86% of patients (Baek et al. 2014). Also, medication changes owing to adverse drug effects, lack of response, and phase changes frequently occur during the course of treatment for BDs (Yatham et al. 2013; Baek et al. 2014; Arvilommi et al. 2010). Given the complexity of pharmacotherapy, the long-term effect of an isolated mood stabilizer is quite difficult to delineate. In addition, the effects of a single medication given alone would be hard to generalize to real-world BD treatment. A more global view of treatment effects on the long-term outcome of BD is required.

This study was designed to estimate the clinical response of individuals with BDs to long-term treatment with mood stabilizers. In order to reflect the prescription patterns shown by a recent nationwide survey that includes data from our clinic (Baek et al. 2014), treatment with the most commonly used mood stabilizers, i.e., valproate and lithium, was selected as target mood stabilizer treatment. The overall response rate was retrospectively assessed using the Alda scale based on observational data collected over a period of more than 2 years. This study also aimed to identify the factors associated with treatment response among all of the comprehensive clinical variables investigated.

## Patients and methods

### Subjects

**Fig. 1:**
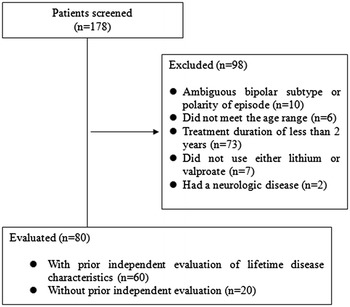
Flow diagram of patient enrollment

Patients who met the DSM-IV criteria for BD-I or BD-II and had received treatment with lithium and/or valproate for more than 2 years between March, 2009 and April, 2015 at the Bipolar Disorder Clinic of the Samsung Medical Center, a tertiary-care university-affiliated hospital, were screened for inclusion in the study. The patients’ ages ranged from 18 to 55 years. Those who had evidence of neurologic disorders or general medical conditions related to mental symptoms were excluded. A total of eighty patients who met the above criteria and agreed to participate in the study were enrolled (Fig. 1). Among those patients, there were 60 participants who were involved in other clinical and genetic studies described elsewhere (Baek et al. 2011, 2016; Yang et al. 2015). This study was approved by the Institutional Review Board of the Samsung Medical Center.

### Assessment of the treatment response

The best treatment (including lithium and/or valproate) was provided to each patient based on treatment guidelines including the Korean Medication Algorithm for Bipolar Disorder 2014 (Shin et al. 2013) and other international guidelines (Yatham et al. 2013; Goodwin 2009), clinicians’ experience, and patients’ special concern on expected adverse effects. Long-term response to the treatment was evaluated through retrospective chart review. When possible, additional information was directly obtained from the patients during their outpatient departmental visits. Assessments were performed using the Alda scale (Grof et al. 2002). The Alda scale consists of two criteria, i.e., rating of the association between clinical improvement and treatment (Criterion A) and rating of the degree of the causal relationship between clinical improvement and prophylactic treatment (Criterion B). The total score was obtained by subtracting the B score from the A score. Two research psychiatrists (SWA and KSH) and the clinician who saw each patient (KSH, JHB, YC, S-YY, or SWA) independently reviewed the hospital records and came to a consensus on the treatment response.

### Assessment of clinical characteristics

For all of the subjects, the current mood state of the subjects was assessed using the clinical global impressions scale for use in bipolar illness (CGI-BP) (Spearing et al. 1997). Predominant polarity was assessed according to the criteria proposed by Colom and colleagues (2006).

For 60 subjects (participants in the previous studies conducted by the authors), comprehensive disease characteristics had previously been evaluated before the present assessment of treatment response. The evaluation was performed through a direct interview using the revised version of the Korean version of the Diagnostic Interview for Genetic Studies (Joo et al. 2004), and is described in detail elsewhere (Baek et al. 2011, 2016; Yang et al. 2015). The rated variables cover age at onset and course of mood episodes, manifested symptoms, and comorbid psychiatric conditions on a lifetime basis.

### Statistical analysis

Patients were classified into good responders and moderate/poor responders as defined based on frequentist mixture analysis using the total Alda scale score. The analysis showed a best-fit theoretical model of two components (AIC = 374.1; BIC = 383.6) (Additional file 1: Table S1), and a suggested cut-off point at a total score of 4.5. Therefore, a total score 5 or higher was defined as a good response and a score of 4 or lower was defined as a moderate/poor response.

Comparison of demographic and clinical variables between the two groups was performed using a Chi-square test (or Fisher’s exact test) for categorical data, and a *t* test for continuous variables. Probability (*p*) values less than 0.05 were considered statistically significant. The same comparisons between good vs. moderate and poor responders were also applied to the BD-I subgroup. All statistical analyses were done with IBM SPSS version 23.0.

## Results

### Pharmacotherapy and treatment response

**Fig. 2:**
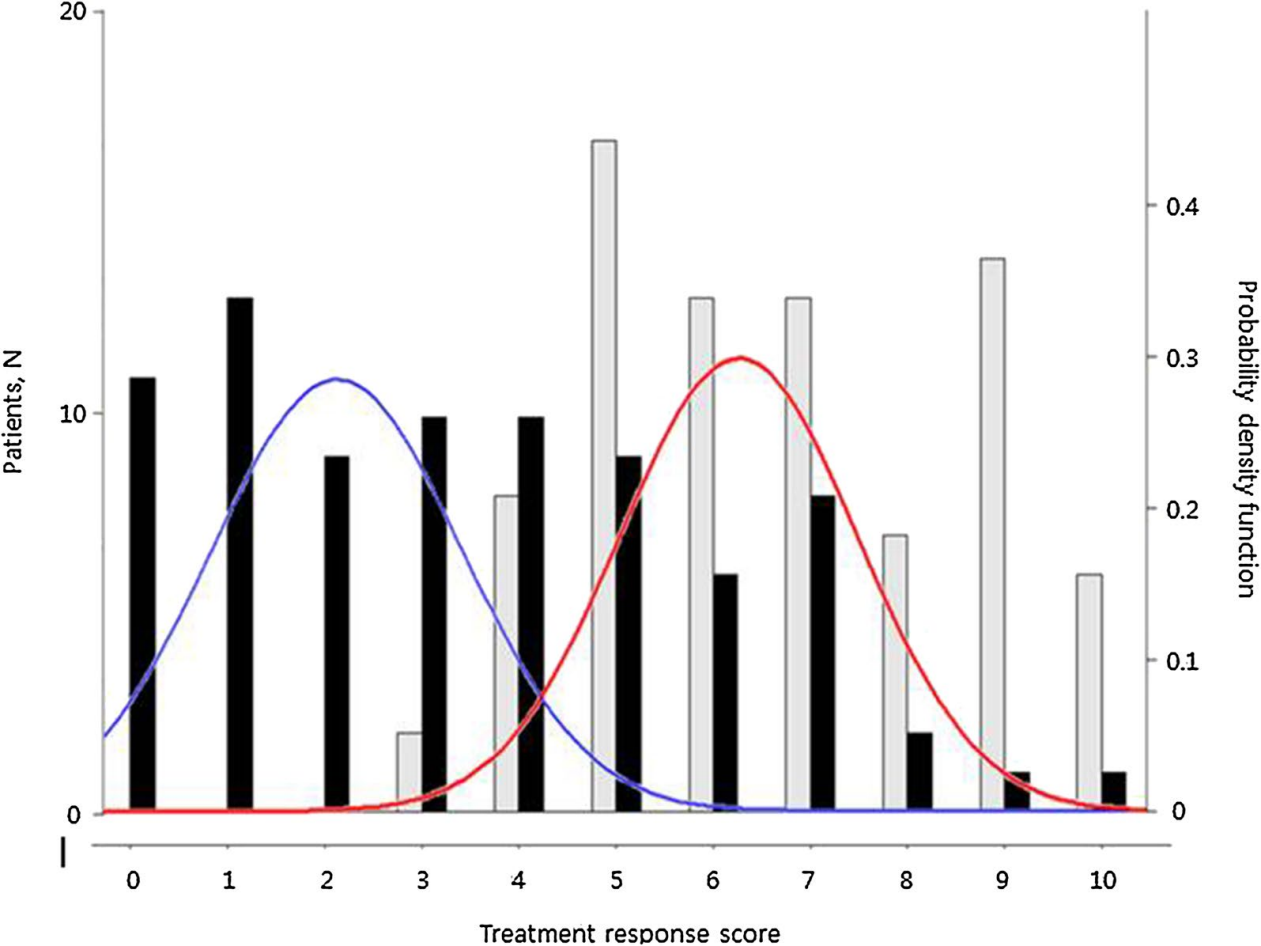
Empirical and theoretical distributions of the treatment response score. This *figure* shows a histogram of total score (*black*) and A score (*gray*) with two density plots of the total score for the two subpopulations. The subpopulations indicate good responders (*red*) and moderate/poor responders (*blue*) as assessed by total Alda scale scores identified by the Bayesian minimum message length method

Among all 80 subjects, 50 (62.5%) received valproate, 19 (23.8%) received lithium, and 11 (13.8%) received both lithium and valproate. The mean duration of medication was 71.7 (SD = 34.1, range: 25–142) months. The total and A scores of the Alda scale are shown in Fig. 2. The mean total score was 3.4 (SD = 2.5), and the mean A score was 6.7 (SD = 1.9). Based on frequentist mixture analysis of the total score, 27 (33.8%) and 53 (66.2%) patients were classified into the good response and moderate/poor response groups, respectively. All of the subjects had received treatment for 2 or more years and showed adequate compliance during the observation period, with more than 80% levels in the therapeutic range. Patients may have been prescribed multiple drugs, and quetiapine was the most frequently prescribed adjunct medication. Additional use of antipsychotics, antidepressants, and other mood stabilizers at the time of the current assessment is summarized in Table 1. As expected, moderate/poor responders received more adjunctive medicines than did good responders. Polypharmacy of mood stabilizers including atypical antipsychotics (except for quetiapine of daily dose of 50 mg or less which is usually applied for insomnia control) was popular in both good responders (*N* = 14, 51.9%) and moderate/poor responders (*N* = 43, 81.1%).

**Table 1:** Current use of adjunct medications: antipsychotics, antidepressants, and other mood stabilizers

Category	Drug	Good responders (*N* = 27)	Moderate/poor responders (*N* = 53)
Antipsychotics, *N* (%)	Quetiapine under 50 mg	0 (0.0)	4 (7.5)
	Quetiapine over 50 mg	5 (18.5)	16 (30.2)
	Risperidone	1 (3.7)	8 (15.1)
	Olanzapine	4 (14.8)	5 (9.4)
	Ziprasidone	0 (0.0)	3 (5.7)
	Aripiprazole	0 (0.0)	9 (17.0)
	Paliperidone	0 (0.0)	2 (3.8)
	Clozapine	1 (3.7)	3 (5.7)
	Amisulpride	1 (3.7)	1 (1.9)
	Blonanserin	1 (3.7)	1 (1.9)
Antidepressants, *N* (%)	Any	0 (0.0)	0 (0.0)
Other mood	Lamotrigine	4 (14.8)	4 (7.5)
Stabilizers, *N* (%)	Carbamazepine	0 (0.0)	1 (1.9)
Mood stabilizer polypharmacy, *N* (%)^a^		14 (51.9)	43 (81.1)
Total number of mood stabilizers, mean (SD)^a^		1.7 (0.8)	2.2 (0.8)

At the time of the assessment of treatment response

^a^Including index medications, atypical antipsychotics, and other mood stabilizers; for quetiapine, daily dose of 50 mg or less which is usually applied for insomnia control was excluded

### Comparison of demographic and disease course characteristics

Table 2 shows the demographic characteristics and clinical course of good responders and moderate/poor responders. Sex and age were not significantly different between the two groups. There were no statistically significant group differences in the age at onset, subtype diagnosis (BD-I vs. BD-II), polarity at the first episode, current smoking status, predominant polarity, number of depressive/(hypo)manic episodes, and family history of psychiatric disorders and mood disorders (in second-degree relatives). Compared to good responders, moderate/poor responders experienced a greater number of mixed episodes before taking their current mood stabilizers (*p* = 0.026). The type of index mood stabilizer was not statistically different between groups, but a much higher rate of combination of lithium and valproate was observed in the moderate/poor responders compared to the good responders (18.9 vs. 3.7%). In terms of efficacy of specific medications, the mean total score of the Alda scale was not significantly different (*t* = −1.423, *p* = 0.159) between valproate users (4.0 ± 2.4) and lithium users (3.0 ± 2.7). Lithium and valproate combination group shows lower mean total score of the Alda scale (1.45 ± 1.8). In the three-group comparison, an overall difference (*p* = 0.008) with a significant difference between the combination group and valproate group (*p* = 0.011, Scheffe’s methods) was observed.

**Table 2:** Comparison of demographic data and clinical course between good responders and moderate/poor responders (*N* = 80)

Variables	Good responders (*N* = 27)	Moderate/poor responders (*N* = 53)	Group difference	
			Statistics	*p* value
Sex, male, *N* (%)	10 (37.0)	13 (24.5)	*χ* ^2^ = 1.366	0.299
Age at study entry, years, mean (SD)	37.0 (9.1)	34.5 (7.3)	*t* = −1.368	0.175
Age at onset, years, mean (SD)^a^	24.9 (8.7)	23.3 (6.3)	*t* = −0.789	0.435
Duration of illness until using the index mood stabilizer, years, mean (SD)	6.07 (4.2)	5.42 (5.5)	*t* = −0.551	0.583
Diagnosis, BD-I, *N* (%)	21 (77.8)	44 (83.0)	*χ* ^2^ = 0.323	0.570
Index medication, *N* (%)			FE	0.143
Lithium	6 (22.2)	13 (24.5)		
Valproate	20 (74.1)	30 (56.6)		
Both lithium and valproate	1 (3.7)	10 (18.9)		
Duration of index mood stabilizer treatment, months, mean (SD)	73.0 (36.4)	71.0 (33.1)	*t* = −0.249	0.804
Polarity at the first episode, (hypo)manic, *N* (%)	13 (48.2)	28 (52.8)	*χ* ^2^ = 0.157	0.692
Predominant polarity^a^			*χ* ^2^ = 0.161	0.922
Manic predominant	8 (32.0)	18 (35.3)		
Depressive predominant	5 (20.0)	11 (21.6)		
Undetermined	12 (48.0)	22 (43.1)		
Number of episodes before using index mood stabilizer, mean (SD)				
Major depressive episodes	1.4 (1.1)	1.2 (1.3)	*t* = −0.630	0.531
Manic episodes^b^	1.3 (1.2)	0.9 (0.8)	*t* = −1.284	0.207
Hypomanic^b^	0.4 (0.8)	0.1 (0.3)	*t* = −1.994	0.056
Mixed episodes^b^	0.12 (0.3)	0.5 (1.2)	*t* = 2.278	0.026
Family history of psychiatric disorders, *N* (%)^c^	16 (59.3)	32 (60.4)	*χ* ^2^ = 0.009	0.923
Family history of mood disorders, *N* (%)^d^	8 (29.6)	20 (37.7)	*χ* ^2^ = 0.517	0.472
Number of hospitalization before using index mood stabilizer treatment, mean (SD)	1.4 (1.2)	1.6 (1.2)	*t* = 1.849	0.622
Current smoker, *N* (%)	5 (20.8)	8 (21.6)	*χ* ^2^ = 0.005	0.941
CGI-BP^e^, severity of overall bipolar illness	1.6 (0.8)	3.2 (0.9)	*t* = 7.876	0.000

*BD-I* bipolar I disorder, *FE* Fisher’s exact test, *SD* standard deviation

^a^ *N* = 76

^b^Equal variance not assumed

^c^Evaluated in second-degree relatives

^d^Evaluated in second-degree relatives, including those with major depressive disorders and bipolar disorders

^e^Clinical global impressions scale for use in bipolar illness

### Comparison of symptom profiles of mood episodes

Lifetime-based symptom profiles of mood episodes are described in Additional file 1: Table S2. When considering the symptoms of (hypo)manic episodes, delusion was much more frequent in moderate/poor responders (72.2%) than in good responders (37.5%) (*p* = 0.008). Other symptoms including elevated mood, irritability, grandiosity, decreased sleep need, talkativeness, flight of idea, distractibility, hyperactivity, excessive involvement in activity, and hallucination were observed at similar rates in both groups. Among symptoms of depressive episodes, only ‘appetite change’ showed a significant between-group difference (*p* = 0.035). Increased appetite was observed only in moderate/poor responders (7 subjects, 19.4%), and appetite loss was more frequent in good responders (63.6%) than in moderate/poor responders (36.1%). The other symptoms which we investigated, i.e., depressed mood, insomnia, hypersomnia, agitation, retardation, apathy, loss of energy, guilty feeling, low self-esteem, suicidal ideation, indecisiveness, delusion, and hallucination did not show any difference between the two groups.

### Comparison of comorbid psychiatric disorders and conditions

While moderate/poor responders had a lifetime co-morbidity of any anxiety disorder of 25%, anxiety disorders were not observed in good responders (*p* = 0.008) (Additional file 1: Table S3). The co-morbidity rate of other psychiatric conditions observed in the current subjects, including alcohol/substance-related disorders, eating disorders (anorexia and/or bulimia nervosa), hyperthymic temperament, premenstrual syndrome, and history of suicidal attempts, did not show significant between-group differences.

### Analyses of possible confounding factors

Considering that the preference for a specific mood stabilizer (lithium, valproate, or a combination of the two) given a specific baseline condition might affect the results of the main analyses, we compared demographic and clinical variables among the three groups divided by medication type (Additional file 1: Tables S4, S5). A between-group difference was observed only in subtype diagnosis (BD-I vs. BD-II). Therefore, we additionally performed the same analyses only in BD-I (*N* = 65) patients. A previous history of mixed episodes (*p* = 0.015) and delusion during manic phases (*p* = 0.003) were again identified as being associated with a worse response (Additional file 1: Tables S6, S7).

## Discussion

In this retrospective investigation of the clinical response of patients with BDs to long-term (2 years or more) treatment with valproate and/or lithium, one-third of the patients were good responders, with a total Alda score of 5 or higher. When our analysis excluded patients receiving both valproate and lithium, we did not find any significant differences in the long-term clinical effects between the two drugs. Previous experience with mixed episodes (according to DSM-IV criteria), delusions during manic episodes, appetite increase during depressive episodes, and comorbid anxiety disorders were related to a worse response.

As the present study does not focus on the effects and associated factors of a single drug, direct comparison of clinical response between the current study and previous studies, most of which investigated the effects of single mood stabilizers in isolation, would be difficult. In order to explore long-term response in a naturalistic clinical setting, we felt it necessary to consider medication changes and combinations of different mood stabilizers during the course of treatment. Therefore, we selected valproate, lithium, and a combination of the two drugs as a single target treatment. In the current study, valproate and lithium seemed to be associated with similar long-term responses. In addition, the supplementary analysis showed that baseline demographic and clinical features did not affect selection of one drug over another, except that there was a preference for valproate in BD-II. Valproate and lithium are the most frequently used medications in our clinic and in other mood disorder clinics in tertiary-care hospitals in Korea (Baek et al. 2014). According to a report from the Stanley Foundation Treatment Outcome Network (Post et al. 2010), these two drugs are the most frequently prescribed medications at the time of improvement and have high overall success rates in outpatients treated for BDs.

Although several studies have investigated the response to mood stabilizers using the Alda scale, they used different response criteria. In a previous report by Garnham and colleagues (2007), which defined the ‘full-responder’ group as those with a total score of 7 or higher, the rate of full response was 30% in lithium users and 13% in valproate users. In the ConLiGen study (Manchia et al. 2013), which used the same criteria, 33% of lithium-treated patients were classified as ‘full responders.’ When adopting the ConLiGen criteria, the full-responder rate was just 15.0% in our study (12 out of 80 patients). The mean total score was also lower in the current study (3.4 ± 2.5) compared to in the ConLiGen studies, i.e., 4.4 ± 3.1 in their initial clinical report (Manchia et al. 2013), and 4.3 ± 3.3 and 3.9 ± 3.0 in their genome-wide association study (Hou et al. 2016). However, the mean A score of the current subjects (6.7 ± 1.9) was higher than that of the ConLiGen study subjects, which ranged from 6.0 to 6.4 (Hou et al. 2016; Manchia et al. 2013). This indicates a higher B score in the current subjects. The B score reflects baseline clinical characteristics that could affect the true causal relationship between treatment and outcome, including previous mood episodes, treatment duration, compliance, and additional medication. One prominent feature of our sample related to B score is a high rate (76.3%) of psychotic features that could result in the usage of additional antipsychotic medications.

To identify the clinical factors associated with long-term mood stabilizer treatment, we investigated a variety of baseline characteristics, including disease onset and course, symptoms of episodes, and comorbidities. Worse treatment response in patients with more previous mixed episodes, delusions during manic episodes, and appetite increase during depressive episodes was observed not only in the main analysis of all subjects but also in the supplementary analysis of BD-I patients alone. Analysis of comorbid psychiatric conditions could be performed only in the overall sample because of the small sample size of the BD-I group, considering the low rates of comorbidities. Anxiety disorders were only observed in the moderate/poor responders. Although study designs and target drugs differ between studies, the current results are roughly in agreement with previous reports of predictors of treatment response to mood stabilizers. Mixed episodes predicted poor long-term response in a study on lithium (Backlund et al. 2009) and a study on multiple mood stabilizers (Peselow et al. 2015). Psychotic features were also reported as a predictive factor of a poor response to lithium (Kleindienst et al. 2005; Backlund et al. 2009; Pfennig et al. 2010; Silva et al. 2016). According to the current results, among psychotic symptoms, delusions during manic episodes were specifically associated with worse responses. Increase in appetite is a major symptom of atypical depression, and atypical features of depression were reported to be associated with a greater rate of psychiatric comorbidities, increased distress, suicidal ideation, and disability, all of which might lead to negative treatment outcomes (Sadock et al. 2014; Matza et al. 2003). To the best of our knowledge, this is the first study to report on the atypical symptoms in depressive episodes as a predictor of poor long-term mood-stabilizer response in BDs. Various comorbid disorders were expected to occur and are known to be associated with poor response in BD patients (Kleindienst et al. 2005; Kliwicki 2014; Rybakowski 2014), and a concordant finding was detected only for anxiety disorders in the current study. In the case of other comorbid conditions, including eating disorders and alcohol/substance-related disorders, the small sample size might have limited our ability to detect associations. Other reported predictors of response, i.e., age at onset, a high number of previous hospitalizations, rapid cycling, and hyperthymic temperament (Kleindienst et al. 2005; Rybakowski et al. 2013), did not show a significant association in the current study, and need to be analyzed in future studies with larger sample sizes. Regarding the controversial results of previous studies on BD-I vs. BD-II (Kleindienst et al. 2005; Garnham et al. 2007; Rybakowski 2014), the current data could not generate any conclusions owing to the extremely skewed use of valproate in BD-II.

This study has several limitations. First, because of the relatively small number of subjects, false negative results are to be expected. The statistical power may be particularly limited to detect differences in patients taking lithium vs. valproate. A large number of patients in the clinic were excluded because they had not taken the index medications for more than 2 years. Second, as this is a retrospective study, a conclusive causal relationship could not be determined between clinical factors and poor response rates. Third, in this naturalistic observational study, many uncontrolled confounding variables might decrease the assay sensitivity. Choice of medication (valproate vs. lithium) and diagnosis of BD-I vs. BD-II were considered as possible confounding factors in the supplementary analyses, whereas the effects of other potential confounding variables, such as medication dose or plasma level, adjunctive medications, and non-pharmacologic treatment, were not excluded. In the comparison of long-term response between drugs, there might be additional confounding variables affecting the selection of a specific mood stabilizer. Fourth, lack of use of classical longitudinal illness metrics, e.g., time to recurrence/recovery is also a limitation of this study considering difficulties in direct comparison of the current results with previous studies. Finally, as all of the subjects were Korean patients, the current results may have limited generalizability to other populations.

This study also has the following strength. It is a naturalistic observational study that reflects a real-world clinical setting. Although retrospective evaluation was performed, reliable assessment of treatment response is expected considering the long follow-up period (at least 2 years) at a single institute and the involvement of the treating clinicians in outcome assessment. In addition, baseline disease characteristics and clinical variables were independently assessed in previous studies by the authors before the current assessment of treatment response.

## Conclusions

This study shows an overall outcome and response rate of BDs to long-term standard treatment using valproate and/or lithium. This study adds additional evidence that mixed and psychotic features and comorbid anxiety disorders are associated with poor treatment response in patients with BDs. It also identifies specific symptoms (increased appetite during depressive episodes and delusions during manic episodes) as novel candidate predictors of long-term mood stabilizer treatment.

## Additional file

**Additional file 1: Table S1.** Model fitting evaluation of the total Alda scale score. **Table S2.** Comparison of lifetime symptom profiles of mood episodes between good responders and moderate/poor responders (N = 60). **Table S3.** Comparison of the lifetime prevalence of co-morbid psychiatric illnesses between good responders and moderate/poor responders (N = 60). **Table S4.** Comparison of baseline characteristics by medication (N = 80). **Table S5.** Comparison of symptom profiles by medication (N = 60). **Table S6.** Comparison of demographic data and clinical course between good responders and moderate/poor responders in patients with bipolar I disorder (N = 65). **Table S7.** Comparison of symptom profiles between good responders and moderate/poor responders (N = 49).
