# Supplementary material for: Long-term response to mood stabilizer treatment and its clinical correlates in patients with bipolar disorders: a retrospective observational study
Source: Int J Bipolar Disord. 2017 Jul 9;5:24. doi: 10.1186/s40345-017-0093-5 (PMC5502006; doi:10.1186/s40345-017-0093-5)
Supplement: Supplementary file 1 — Additional file 1: Table S1. Model fitting evaluation of the total Alda scale score. Table S2. Comparison of lifetime symptom profiles of mood episodes between good responders and moderate/poor responders (N = 60). Table S3. Comparison of the lifetime prevalence of co-morbid psychiatric illnesses between good responders and moderate/poor responders (N = 60). Table S4. Comparison of baseline characteristics by medication (N = 80). Table S5. Comparison of symptom profiles by medication (N = 60). Table S6. Comparison of demographic data and clinical course between good responders and moderate/poor responders in patients with bipolar I disorder (N = 65). Table S7. Comparison of symptom profiles between good responders and moderate/poor responders (N = 49). [file 40345_2017_93_MOESM1_ESM.docx]

Supplementary Table S1. Model fitting evaluation of the total Alda scale score

|  | Total score |  |
| --- | --- | --- |
| Number of group | AIC | BIC |
| 2 | 374.1 | 383.6 |
| 3 | 378.1 | 392.4 |
| 4 | 378.8 | 397.9 |
| 5 | 379.1 | 402.9 |
| 6 | 383.1 | 411.7 |
| 7 | 382.8 | 416.2 |
| 8 | 386.8 | 425.0 |
| 9 | 390.0 | 432.8 |
| 10 | 394.1 | 441.8 |

Supplementary Table S2. Comparison of lifetime symptom profiles of mood episodes between good responders and moderate/poor responders (N=60)

| In (hypo) manic episode | Good responders  (N=24) | Moderate/poor responders (N=36) | Group difference | |
| --- | --- | --- | --- | --- |
|  | Present, N (%) | Present, N (%) | χ2 | p-value |
| Elevated mood | 22 (91.7) | 31 (86.1) | FE | 0.691 |
| Irritability | 23 (95.8) | 32 (88.9) | FE | 0.639 |
| Grandiosity | 16 (66.7) | 25 (69.4) | 0.051 | 0.821 |
| Decreased sleep need | 19 (79.2) | 28 (77.8) | 0.016 | 0.898 |
| Talkativeness | 22 (91.7) | 30 (83.3) | FE | 0.457 |
| Flight of idea | 17 (70.8) | 30 (83.3) | 1.326 | 0.250 |
| Distractibility | 19 (79.2) | 27 (75.0) | 0.140 | 0.709 |
| Hyperactivity | 21 (87.5) | 33 (91.7) | FE | 0.675 |
| Excessive involvement in activity | 16 (66.7) | 22 (61.1) | 0.191 | 0.662 |
| Delusion | 9 (37.5) | 26 (72.2) | 7.143 | 0.008 |
| Hallucination | 5 (20.8) | 9 (25.0) | 0.140 | 0.709 |
| In depressive episode | Good responders (N=22) | Moderate/poor responders (N=36) | Group difference | |
|  | Present, N (%) | Present, N (%) | χ2 | p-value |
| Depressed mood (vs. Anhedonia) | 19 (86.4) | 30 (83.3) | FE | 1.000 |
| Appetite change |  |  | FE | 0.035 |
| no appetite change | 8 (36.4) | 16 (44.4) |  |  |
| decreased | 14 (63.6) | 13 (36.1) |  |  |
| increased | 0 (0.0) | 7 (19.4) |  |  |
| Insomnia | 10 (45.5) | 15 (41.7) | 0.080 | 0.777 |
| Hypersomnia | 6 (27.3) | 11 (30.6) | 0.071 | 0.790 |
| Agitation | 5 (22.7) | 12 (33.3) | 0.741 | 0.389 |
| Retardation | 13 (59.1) | 19 (52.8) | 0.220 | 0.639 |
| Apathy | 18 (81.8) | 26 (72.2) | 0.687 | 0.407 |
| Fatigue or loss of energy | 19 (86.4) | 25 (69.4) | 2.135 | 0.144 |
| Guilty feeling | 7 (31.8) | 13 (36.1) | 0.111 | 0.739 |
| Low self-esteem | 9 (40.9) | 20 (55.6) | 1.172 | 0.279 |
| Suicidal ideation | 13 (59.1) | 23 (63.9) | 0.134 | 0.715 |
| Indecisiveness | 16 (72.7) | 28 (77.8) | 0.190 | 0.663 |
| Delusion | 5 (22.7) | 13 (36.1) | 1.143 | 0.285 |
| Hallucination | 2 (9.1) | 4 (11.1) | FE | 1.000 |

FE – Fisher’s exact test

Supplementary Table S3. Comparison of the lifetime prevalence of co-morbid psychiatric illnesses between good responders and moderate/poor responders (N=60)

|  | Good responders  (N=24) | Moderate/poor responders  (N=36) | Group difference | |
| --- | --- | --- | --- | --- |
| Comorbid diagnosis | N (%) | N (%) | χ2 | p-value |
| Any co-morbid psychiatric illness | 3 (12.5) | 11 (30.6) | 2.624 | 0.105 |
| Any anxiety disorder | 0 (0.0) | 9 (25.0) | FE | 0.008 |
| Panic disorder | 0 (0.0) | 3 (8.3) | FE | 0.268 |
| Phobia | 0 (0.0) | 3 (8.3) | FE | 0.268 |
| Obsessive-compulsive disorder | 0 (0.0) | 6 (16.7) | FE | 0.072 |
| Alcohol/substance-related disorder ^a^ | 3 (12.5) | 2 (5.4) | FE | 0.373 |
| Eating disorder | 1 (4.2) | 2 (5.6) | FE | 1.000 |
| Hyperthymic temperament | 1 (4.2) | 4 (11.1) | FE | 0.639 |
| Suicidal attempt | 7 (29.2) | 10 (27.8) | 0.014 | 1.000 |
| Premenstrual syndrome ^b^ | 5 (29.4) | 11 (29.0) | FE | 1.000 |

FE – Fisher’s exact test

^a^ N = 61 (24 good responders, 37 moderate/poor responders)

^b^ N = 55 (female patients; 17 good responders, 38 moderate/poor responders)

Supplementary Table S4. Comparison of baseline characteristics by medication (N =80)

|  | Lithium (N=19) | Valproate (N=50) | Both drugs (N=11) | Group difference | | | |
| --- | --- | --- | --- | --- | --- | --- | --- |
|  |  |  |  | Statistics | p-value | |  |
| Sex, male, N(%) | 4 (21.1) | 15 (30.0) | 4 (36.4) | χ^2^=0.899 | 0.638 |  |  |
| Age at study entry, years, mean(SD) | 36.11 (9.6) | 35.40 (7.4) | 33.64 (8.5) | F=0.329 | 0.720 |  |  |
| Age at onset, years, mean(SD) | 24.95 (7.4) | 23.16 (6.6) | 24.91 (9.0) | F=0.567 | 0.569 |  |  |
| Duration of illness until using index mood stabilizer, years, mean(SD) | 6.47 (6.6) | 5.48 (4.6) | 4.91 (3.8) | F=0.395 | 0.675 |  |  |
| Diagnosis, BD-I, N(%) | 19 (100) | 35 (70.0) | 11 (100) | FE | 0.002 |  |  |
|  |  |  |  |  |  |  |  |
| Polarity at the first episode, (hypo)manic, N(%) | 13 (68.4) | 20 (40.0) | 6 (54.5) | χ^2^=4.623 | 0.099 |  |  |
| Number of Episodes before using index mood stabilizer, mean(SD) |  |  |  |  |  |  |  |
| Major depressive episodes | 1.0 (1.1) | 1.4 (1.3) | 0.91 (0.8) | F=1.075 | 0.347 |  |  |
| Manic episodes | 1.1 (0.8) | 1.0 (1.1) | 0.91 (0.9) | F=0.137 | 0.872 |  |  |
| Hypomanic episodes | 0.3 (0.6) | 0.21 (0.6) | 0.18 (0.4) | F=0.186 | 0.831 |  |  |
| Mixed episodes | 0.5 (1.0) | 0.22 (0.5) | 0.82 (2.1) | F=1.931 | 0.152 |  |  |
| Family history of psychiatric disorders, N(%) ^b^ | 13 (68.4) | 29 (58.0) | 6 (54.5) | χ^2^=0.781 | 0.677 |  |  |
| Family history of mood disorders, N(%) ^c^ | 7 (36.8) | 17 (34.0) | 4 (36.4) | χ^2^=0.059 | 0.971 |  |  |
| Number of hospitalization before using index mood stabilizer treatment, mean(SD) | 1.63 (1.30) | 1.46 (1.1) | 1.73 (1.5) | F=0.288 | 0.750 |  |  |

FE, Fisher’s exact test

^a^ Scheffe: valproate > both lithium and valproate (p=0.038)

^b^ Evaluated in second-degree relatives.

^c^ Evaluated in second-degree relatives, including those with major depressive disorders and bipolar disorders

Supplementary Table S5. Comparison of symptom profiles by medication (N=60)

| In (hypo) manic episode | Lithium (N=14) | Valproate (N=36) | Both drugs (N=10) | Group difference | |
| --- | --- | --- | --- | --- | --- |
|  | Present, N (%) | Present, N (%) | Present, N (%) | χ^2^ | p-value |
| Elevated mood | 13 (92.9) | 32 (88.9) | 8 (80.0) | FE | 0.725 |
| Irritability | 14 (100.0) | 31 (86.1) | 10 (100.0) | FE | 0.262 |
| Grandiosity | 12 (85.7) | 25 (69.4) | 4 (40.0) | FE | 0.076 |
| Decreased sleep need | 12 (85.7) | 27 (75.0) | 8 (80.0) | FE | 0.904 |
| Talkativeness | 12 (85.7) | 32 (88.9) | 8 (80.0) | FE | 0.759 |
| Flight of idea | 12 (85.7) | 28 (77.8) | 7 (70.0) | FE | 0.620 |
| Distractibility | 13 (92.9) | 25 (69.4) | 8 (80.0) | FE | 0.216 |
| Hyperactivity | 13 (92.9) | 32 (88.9) | 9 (90.0) | FE | 1.000 |
| Excessive involvement in activity | 10 (71.4) | 21 (58.3) | 7 (70.0) | 0.974 | 0.614 |
| Delusion | 10 (71.4) | 18 (50.0) | 7 (70.0) | 2.576 | 0.276 |
| Hallucination | 1 (7.1) | 10 (27.8) | 3 (30.0) | FE | 0.265 |
| In depressive episode | Lithium (N=14) | Valproate (N=35) | Both lithium and valproate (N=9) | Group difference | |
|  | Present, N (%) | Present, N (%) | Present, N (%) | χ^2^ | p-value |
| Depressed mood (vs. Anhedonia) | 11 (78.6) | 31 (88.6) | 7 (77.8) | FE | 0.519 |
| Appetite change |  |  |  | FE | 0.854 |
| no appetite change | 5 (35.7) | 14 (40.0) | 5 (55.6) |  |  |
| Appetite loss | 8 (57.1) | 16 (45.7) | 3 (33.3) |  |  |
| Appetite gain | 1 (7.1) | 5 (14.3) | 1 (11.1) |  |  |
| Insomnia | 6 (42.9) | 17 (48.6) | 2 (22.2) | 2.027 | 0.363 |
| Hypersomnia | 4 (28.6) | 12 (34.3) | 1 (11.1) | FE | 0.472 |
| Agitation | 5 (35.7) | 10 (28.6) | 2 (22.2) | FE | 0.846 |
| Retardation | 9 (64.3) | 21 (60.0) | 2 (22.2) | FE | 0.113 |
| Apathy | 10 (71.4) | 28 (80.0) | 6 (66.7) | FE | 0.573 |
| Fatigue_Loss of energy | 10 (71.4) | 28 (80.0) | 6 (66.7) | FE | 0.573 |
| Guilty feeling | 6 (42.9) | 13 (37.1) | 1 (11.1) | FE | 0.266 |
| Low self-esteem | 5 (35.7) | 21 (60.0) | 3 (33.3) | FE | 0.188 |
| Suicidal ideation | 6 (42.9) | 26 (74.3) | 4 (44.4) | 5.601 | 0.061 |
| Indecisiveness | 10 (71.4) | 28 (80.0) | 6 (66.7) | FE | 0.573 |
| Delusion | 4 (28.6) | 10 (28.6) | 4 (44.4) | FE | 0.663 |
| Hallucination | 0 (0.0) | 4 (11.4) | 2 (22.2) | FE | 0.166 |

FE, Fisher’s exact test

Supplementary Table S6. Comparison of demographic data and clinical course between good responders and moderate/poor responders in patients with bipolar I disorder (N =65)

|  | Good responders (N=21) | Moderate/poor responders (N=44) | Group difference | |
| --- | --- | --- | --- | --- |
| Variable |  |  | Statistics | p-value |
| Sex, male, N(%) | 13 (61.9) | 31 (70.5) | χ^2^=0.475 | 0.491 |
| Age at study entry, years, mean (SD) | 36.5 (9.1) | 33.3 (6.8) | t=-1.623 | 0.110 |
| Age at onset, years, mean (SD) ^a^ | 24.2 (8.6) | 22.9 (6.0) | t=-0.617 | 0.542 |
| Duration of illness until using index mood stabilizer, years, mean(SD) | 6.5 (4.1) | 4.9 (5.1) | t=-1.221 | 0.227 |
| Index medication, N(%) |  |  | χ^2^=3.663 | 0.160 |
| Lithium | 6 (28.6) | 13 (29.5) |  |  |
| Valproate | 14 (66.7) | 21 (47.7) |  |  |
| Both lithium and valproate | 1 (4.8) | 10 (22.7) |  |  |
| Duration of index mood stabilizer treatment, months, mean (SD) | 68.8 (36.0) | 69.9 (32.4) | t=0.230 | 0.818 |
| Polarity at the first episode, (hypo)manic, N(%) | 14 (66.7) | 24 (54.5) | χ^2^=0.860 | 0.354 |
| Number of Episodes before using mood stabilizer, mean(SD) |  |  |  |  |
| Major depressive episodes | 1.3 (1.2) | 1.0 (0.9) | t=-1.081 | 0.284 |
| Manic episodes ^a^ | 1.62 (1.2) | 1.07 (0.8) | t=-1.968 | 0.059 |
| Hypomanic episodes ^a^ | 0.26 (0.6) | 0.09 (0.3) | t=-1.265 | 0.219 |
| Mixed episodes ^a^ | 0.05 (0.2) | 0.55 (1.3) | t=2.511 | 0.015 |
| Family history of psychiatric disorders, N(%) ^b^ | 11 (52.4) | 25 (56.8) | χ^2^=0.113 | 0.736 |
| Family history of mood disorders, N(%) ^c^ | 4 (19.0) | 16 (36.5) | χ^2^=2.001 | 0.157 |
| Number of hospitalization before using index mood stabilizer treatment, mean(SD) | 1.57 (1.1) | 1.81 (1.2) | T=0.761 | 0.450 |

BP-I**,** Bipolar I disorder; FE, Fisher’s exact test; SD, standard deviation

^a^ Equal variance not assumed

^b^ Evaluated in second-degree relatives.

^c^ Evaluated in second-degree relatives, including those with major depressive disorders and bipolar disorders

Supplementary Table S7. Comparison of symptom profiles between good responders and moderate/poor responders (N=49)

| In (hypo) manic episode | Good responders  (N=19) | Moderate/poor responders (N=30) | Group difference | |
| --- | --- | --- | --- | --- |
|  | Present, N (%) | Present, N (%) | χ^2^ | p-value |
| Elevated mood | 25 (83.3) | 17 (89.5) | FE | 0.691 |
| Irritability | 19 (100.0) | 30 (100.0) | - | - |
| Grandiosity | 14 (73.7) | 21 (70.0) | 0.077 | 0.781 |
| Decreased sleep need | 18 (94.7) | 25 (83.3) | FE | 0.384 |
| Talkativeness | 17 (89.5) | 24 (80.0) | FE | 0.458 |
| Flight of idea | 14 (73.7) | 25 (83.3) | FE | 0.480 |
| Distractibility | 17 (89.5) | 24 (80.0) | FE | 0.458 |
| Hyperactivity | 16 (84.2) | 27 (90.0) | FE | 0.665 |
| Excessive involvement in activity | 13 (68.4) | 19 (63.3) | 0.133 | 0.715 |
| Delusion | 9 (47.4) | 26 (86.7) | 8.803 | 0.003 |
| Hallucination | 5 (26.3) | 9 (30.0) | 0.077 | 0.781 |
| In depressive episode | Good responders  (N=17) | Moderate/poor responders (N=30) | Group difference | |
|  | Present, N (%) | Present, N (%) | χ^2^ | p-value |
| Depressed mood (vs. Anhedonia) | 14 (82.4) | 24 (80.0) | FE | 1.000 |
| Appetite change |  |  | FE | 0.121 |
| no appetite change | 7 (41.2) | 12 (40.0) |  |  |
| decreased | 10 (58.8) | 12 (40.0) |  |  |
| increased | 0 (0.0) | 6 (20.0) |  |  |
| Insomnia | 5 (29.4) | 12 (40.0) | 0.527 | 0.468 |
| Hypersomnia | 6 (35.3) | 8 (26.7) | 0.386 | 0.534 |
| Agitation | 2 (11.8) | 10 (33.3) | FE | 0.165 |
| Retardation | 12 (70.6) | 15 (50.0) | 1.882 | 0.170 |
| Apathy | 14 (82.4) | 21 (70.0) | FE | 0.492 |
| Fatigue or loss of energy | 14 (82.4) | 19 (63.3) | 1.877 | 0.171 |
| Guilty feeling | 5 (29.4) | 10 (33.3) | 0.077 | 0.782 |
| Low self-esteem | 6 (35.3) | 17 (56.7) | 1.984 | 0.159 |
| Suicidal ideation | 8 (47.1) | 17 (56.7) | 0.402 | 0.526 |
| Indecisiveness | 12 (70.6) | 22 (73.3) | FE | 1.000 |
| Delusion | 4 (23.5) | 12 (40.0) | 1.311 | 0.252 |
| Hallucination | 2 (11.8) | 3 (10.0) | FE | 1.000 |

FE, Fisher’s exact test
